# Differential IL-1 signaling induced by BMPR2 deficiency drives pulmonary vascular remodeling

**DOI:** 10.1177/2045893217729096

**Published:** 2017-09-22

**Authors:** Josephine Pickworth, Alexander Rothman, James Iremonger, Helen Casbolt, Kay Hopkinson, Peter M. Hickey, Santhi Gladson, Sheila Shay, Nicholas W. Morrell, Sheila E. Francis, James D. West, Allan Lawrie

**Affiliations:** 1 Department of Infection, Immunity & Cardiovascular Disease, University of Sheffield, Sheffield, UK; 2 Vanderbilt Institute, Nashville, TN, USA; 3 Department of Medicine, University of Cambridge, Cambridge, UK

**Keywords:** interleukin-1ß, pulmonary hypertension, inflammation, BMPR-II

## Abstract

Bone morphogenetic protein receptor type 2 (BMPR2) mutations are present in patients with heritable and idiopathic pulmonary arterial hypertension (PAH). Circulating levels of interleukin-1 (IL-1) are raised in patients and animal models. Whether interplay between BMP and IL-1 signaling can explain the local manifestation of PAH in the lung remains unclear. Cell culture, siRNA, and mRNA microarray analysis of RNA isolated from human pulmonary artery (PASMC) and aortic (AoSMC) smooth muscle cells were used. R899X^+/–^ BMPR2 transgenic mice fed a Western diet for six weeks were given daily injections of IL-1ß prior to assessment for PAH and tissue collection. PASMC have reduced inflammatory activation in response to IL-1ß compared with AoSMCs; however, PASMC with reduced BMPR2 demonstrated an exaggerated response. Mice treated with IL-1ß had higher white blood cell counts and significantly raised serum protein levels of IL-6 and osteoprotegerin (OPG) plasma levels recapitulating in vitro data. Phenotypically, IL-1ß treated mice demonstrated increased pulmonary vascular remodeling. IL-1ß induces an exaggerated pulmonary artery specific transcriptomic inflammatory response when BMPR2 signaling is reduced.

Pulmonary arterial hypertension (PAH) is driven by vasoconstriction, inflammatory cell infiltration, vascular cell migration, proliferation, and apoptosis.^1^ Molecular mechanisms regulating PAH pathogenesis are multifactorial involving potassium channels, genetic mutations, serotonin imbalances, estradiol changes, and inflammatory alterations among others.^2,3^

Vascular remodeling in PAH is confined to the lung and patients with the disease do not exhibit alterations in systemic blood pressure or peripheral vascular pathology.^4^ This observation indicates that inherent differences in the cellular behavior in the vascular beds could potentially be one of the possible reasons why global BMPR2 changes only lead to pathogenic remodeling of the pulmonary vasculature while the systemic vessels remain unaltered.

BMPR2 mutations are the primary genetic risk for PAH implicated in over 70% of heritable and 25% of sporadic cases; however, disease penetrance even within families is low (around 15%).^5^ Inflammatory diseases are associated with PAH, and levels of the pro-inflammatory cytokine interleukins (IL)1/6 are increased in PAH.^6^ IL-6 over-expression in vascular cells is enough to induce pulmonary vessel remodeling and PAH in a rodent model.^7^ Inflammation has been shown to be increased in patients and a link with BMPR2 deficiency has been considered.^8^ Previous animal studies have demonstrated that monocrotaline (MCT)-induced PAH is prevented by administration of IL-1 receptor antagonist^9^ and that Apolipoprotein-E knockout mice develop PAH in an IL-1 dependent manner, and via a lung specific putative IL-1R1 receptor.^10^ Interestingly, recent data demonstrated that mice with a heterozygous mutation in BMPR2 have an increased inflammatory response to Lipopolysaccharide.^11^ Further recent literature indicates that the IL1R1/MyD88 signaling pathway is critical to development of animal models of PAH.^12^ We therefore hypothesized that under conditions of reduced BMPR2 expression/signaling, IL-1ß can act as a pulmonary-specific disease-modifying secondary stimulus, thereby demonstrating a direct link between IL-1ß, BMPR2, and disease pathogenesis.

## Methods

### Smooth muscle cell culture and transfection

Human pulmonary artery (hPASMC) (Lonza CC-2581) from three commercial donors and four primary donors, including those with and without disease-related BMPR2 mutations, and human aortic smooth muscle cells (hAoSMC) (Lonza CC-2571) from two donors were maintained in smooth muscle cell media SmGM-2 including bullet kit supplements (Lonza CC-3182). All experiments were performed between passages 4 and 8. Quiescent media (Dulbecco’s modified Eagles medium (Lonza 12-604) with 0.2% (v/v) fetal bovine serum ((FBS) Lonza 14-401) was added for 48 h prior to transfection with siRNA/reporter plasmid (Qiagen Cignal NFkB luciferase reporter) and/or 6-h stimulation with IL-1ß at 10 ng/mL or BMP4 at 20 ng/mL.^13,14^

### RNA extraction

RNA was isolated using Trizol, Direct-zol RNA mini prep kits (Zymo research R2050), and Zymospin column as per manufacturer’s instructions. Eluted RNA quality was assessed using the Agilent Bioanalyzer 2100.

### Microarray and bioinformatic analysis

Three sample RNA pools were run per sub-array with three individual RNA pool sub-arrays per condition. Agilent microarray was performed (Agilent 5190-2305, 5188-5282, 5188-5242, 5188-5327, and RNeasy mini kit, Qiagen 74104) following the labeling protocol (version 6.6, September 2012 Agilent Technologies, G4140-90040) per manufacturer’s instructions and scanned using the Agilent G2565BA scanner.

Data analysis was performed using the Bioconductor package Limma in the programing language “R.” Target files were created containing Agilent G2565BA microarray scanner output files. Background correction, cyclic loess normalization, and averaging of repeats was performed. A linear modeling matrix was built and fitted. Gene lists were filtered discarding those unaltered by IL-1ß in the order of a log2 fold-change of < 1 and for an adjusted *P* value of < 0.05. A pathway analysis functional output was obtained using Signaling Pathway Impact Analysis (SPIA) in R. All was as described in previous papers from our group.^13^ A two-dimensional projection of the microarray expression data was generated using the non-parametric dimensionality reduction. This was achieved using the t-distributed stochastic neighbor embedding (t-SNE) algorithm in the R package Rtse. The resulting t-SNE output was plotted with R package ggplot2. The array data will be deposited in NCBI’s Gene Expression Omnibus.

### Luciferase reporter assay

Following 48 h of incubation with siRNA, reporter plasmid, and stimulants, cells were lysed. Firefly and renila luciferase were read using Promega dual glo assay as per manufacturer’s instructions using a Varioskan Plate reader.

### Real-time polymerase chain reaction of cellular mRNA samples

RNA (n = 9–17 for each condition) was reverse transcribed to cDNA using RNA to cDNA kit (Applied Biosystems 4387406). TaqMan probes for BMPR2 Hs00176148, IL-6 Hs1075666, SOD2 Hs00167309, OPG Hs00917067, VIPR1 Hs00910453 were purchased from Thermo Fisher and run in duplicate. Human ribosomal 18S Hs99999901 was used as control. Relative quantity was calculated using the ΔΔCt method.^15^

### Animal models

Rosa26-rtTA2 × TetO7-Bmpr2R899X mice (called Rosa26-Bmpr2R899X) were used with mutant expression induced by doxycycline as previously described.^16^ Twenty-four Rosa26-Bmpr2R899X transgenic mice and 12 C57 wild-type littermates were fed a Western diet for six weeks and injected with IL-1ß or placebo i.p. once daily for the final four weeks. Mice were assessed for inflammatory activation and PAH phenotype as previously described.^17^ Following six weeks of treatment, the mice were given injectable anesthesia for terminal surgery. Animals underwent full hemodynamic phenotyping, including echocardiography, right and left heart catheterization, blood sampling by cardiac puncture, and a full range of tissues taken and snap frozen. All animal studies were pre-approved by Vanderbilt University Institutional Animal Care and Use Committee.

### Enzyme-linked immunosorbent assay

Mouse serum samples were run using assay DY805 (OPG) and DY206 (IL-6) as per manufacturer’s instructions.

## Results

### IL-1ß stimulation and BMPR2 dysfunction elicit vascular bed-specific transcriptional regulation in smooth muscle cells

**Fig. 1.**
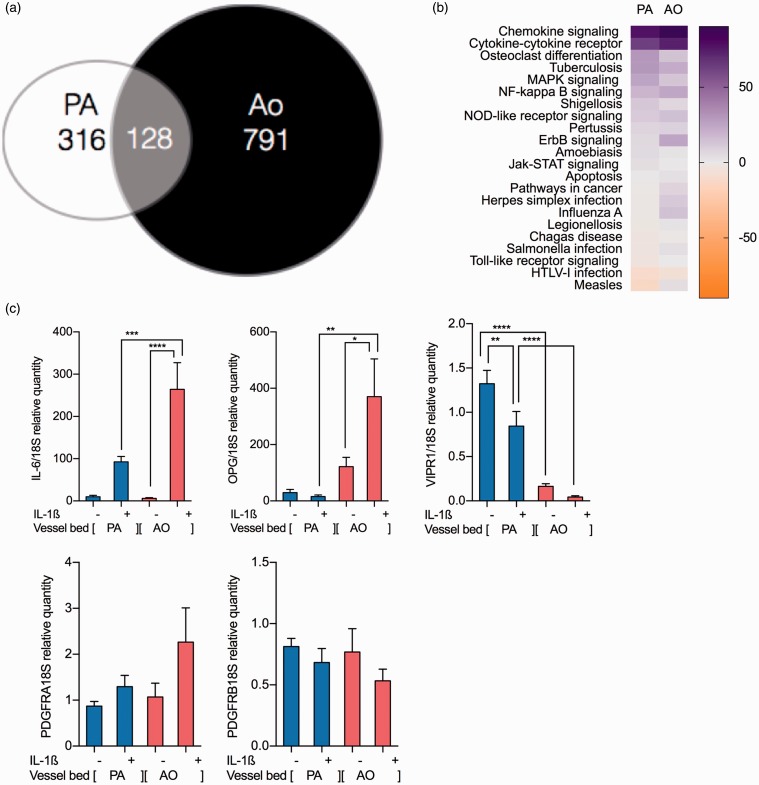
Microarray analysis identifies tissue bed specific changes in of IL-1ß transcriptome. mRNA expression patterns in PASMC and AoSMC (n = 9) (a). Changes in mRNA showing a log2 fold-change of > 1 with false discovery rate (FDR)-adjusted *P* value ≤ 0.05. Pathway analysis was performed using signaling pathway impact analysis (SPIA) pathway analysis software on IL-1ß stimulated in PASMC by using the SPIA package in Limma using programming language R; all gene information from the PASMC arrays are taken into account and cross-referenced against known pathways. Altered pathways discovered using SPIA with significant changes to IL-1ß stimulation in PASMC were compared with the AoMSC IL-1ß responsive pathways changes to generate heatmaps indicating their activation or inhibition in each cell type (b). qRT-PCR validation was performed in PASMC and AoSMC showing increased expression of inflammatory proteins IL-6 and OPG being more pronounced in AoSMC than PASMC; PASMC have a sevenfold higher baseline level of VIPR1 which is reduced to IL-1ß. Receptors also showed PDGF receptors a and b being unaltered to IL-1ß in either cell type. (c) Ordinary one-way ANOVA with multiple comparison by Tukey’s post-test (*P < 0.05, **P < 0.01, ***P < 0.001, ****P < 0.0001).

PAH is a pulmonary arterial-specific disease suggesting that there may be vascular bed-specific transcriptional regulation. A major portion of the vascular disease pathology within the lesions is driven by the proliferation and migration of alpha smooth muscle actin (SMA) positive cells and we therefore sought to characterize and compare the cellular signaling profile in smooth muscle actin positive cells from the pulmonary and aortic smooth muscle cells (PASMC and AoSMC, respectively). In total, 1235 genes were significantly differentially expressed across both cell types in response to IL-ß: 444 in PASMC contrasting with 919 in AoSMCs. Of these genes, 128 overlap in both cell types (Fig. 1a). Subsequent SPIA identified significant differences in the pathways represented by the genes specific to each cell type (SPIA graphs and tables for the genes specific to PA and Ao as Supplemental Fig. 1a and 1b, respectively). Comparison of the altered PASMC pathways highlighted differences in disease relevant pro-migratory and pro-proliferative pathways. “Pathways in Cancer” and infectious disease pathways were altered containing disease-relevant wnt, FADD, and MEK signaling. These pathways were activated in the AoSMC cells, but responses were either inhibited or suppressed in the PASMC (Fig. 1b).

Microarray validation using repeat and separate donor cells was performed by quantitative reverse transcription polymerase chain reaction (qRT-PCR) on selected pathway and disease-relevant genes.^13^ AoSMC demonstrated greater increases in inflammatory genes than PASMC given the same stimulus. All genes analyzed validated array findings in terms of increase or decrease of gene expression, although the degree of alteration varied between platforms. Inflammatory and pro-apoptotic genes, including IL-6 and OPG, are increased in AoSMC to a larger extent than PASMC. However, PASMC demonstrated an increased baseline level of VIPR1 compared to AoSMC but lost expression when stimulated with IL-1ß. Disease-relevant receptors were also measured. There were no changes to IL-1ß in PDGF receptors a or b (Fig. 1c).

### BMPR2 deficiency exaggerates inflammatory activation to IL-1ß stimulation in PASMC but not AoSMC

**Fig. 2.**
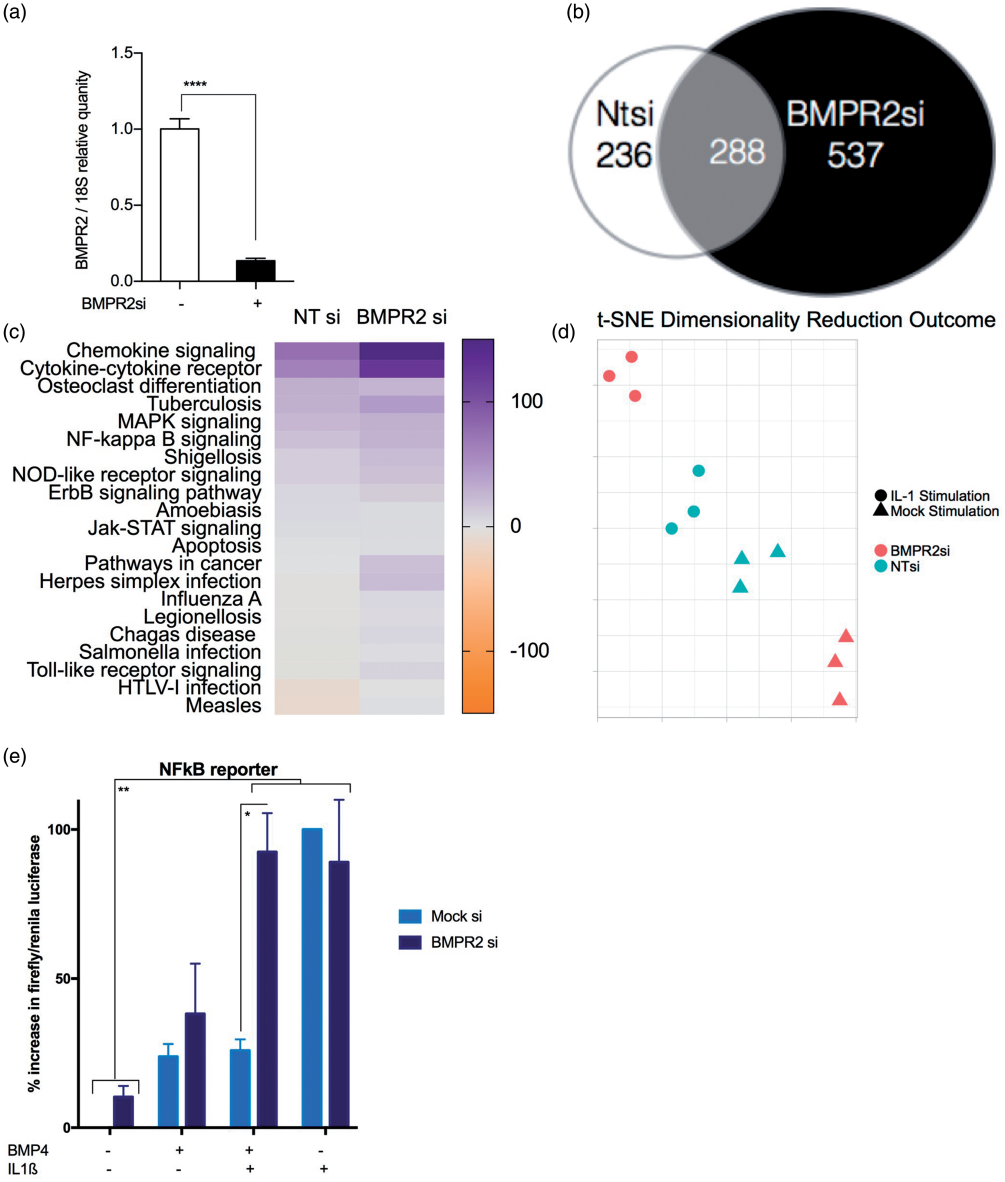
Microarray investigation in PASMC identifies BMPR2 specific changes in IL-1ß transcriptome. qRT-PCR was performed on pooled samples to confirm knockdown prior to arrays (n = 6 for BMPR2si and 3 for NTSi) (a). mRNA expression patterns in PASMC +/– BMPR2 siRNA (n = 9). Ordinary one-way ANOVA with multiple comparisons by Tukey’s post-test (****P < 0.0001). Changes in mRNA showing log2 fold-change of > 1 with adjusted *P* value ≤ 0.05 (b). Altered pathways discovered using SPIA with significant changes to IL-1ß stimulation in PASMCs +/– BMPR2 siRNA. Pathway changes to generate a heatmap indicating their activation or inhibition in each condition (c). t-SNE analysis was conducted on all arrays mock-transfected and BMPR2 si-transfected data-points for dimensionality reduction and shows data with multiple feature correlation in two dimensions (d). PASMC were co-transfected with Cignal NFkB reporter plasmid and NTsiRNA or BMPR2 siRNA and stimulated with BMP4 +/– IL-1ß for 48 h (e). Results were normalized to renila expression in same plasmid and then expressed as percentage of IL-1ß treated positive controls. Two-way ANOVA with Tukey post-test correction (*P < 0.05, **P < 0.01).

The BMP and IL-1ß signaling pathways are linked through the co-localization and co-expression of gene family members (http://www.genemania.com, Supplemental Fig. 2), although the direct influence of one upon the other in PASMC is unknown. We sought to determine the effect of reduced BMPR2 expression in PASMCs using siRNA, and alterations induced by subsequent stimulation with IL-1ß, using whole-genome microarray. Knock-down of BMPR2 was confirmed by RT-PCR (Fig. 2a). Stimulation with IL-1ß resulted in the differential expression of 825 genes on the background of reduced BMPR2 expression compared to 524 in control non-targeting (Ntsi) siRNA-treated cells (Fig. 2b). SPIA highlighted changes in pathway activation in “pathways in cancer” and “rheumatoid arthritis” (SPIA graphs and tables for the genes specific to PA with and without functional BMPR2 as Supplemental Fig. 2a and 2b, respectively). Overall, there were significant increases in the pro-inflammatory, pro-proliferative, and migratory pathways activated, most notably the massive increased activation of the cytokine and chemokine signaling pathways after IL-1ß stimulation in PASMCs where BMPR2 expression is reduced (Fig. 2c). This is reflected in the t-SNE analysis showing that the microarray data clusters into the conditions. In non-targeting silencing RNA-treated PASMC, the IL-1ß makes an alteration in clustering; however, this alteration is exaggerated in the presence on BMPR2 silencing RNA (Fig. 2d).

For further evidence of interactions between the two pathways, NFkB reporter plasmid (Qiagen Cignal NFkB luc reporter assay) was used to assess the changes in IL-1ß signaling through NFkB in the presence and absence of BMP pathway activation. These experiments showed that activation of the BMP signaling pathway by stimulation with BMP4 repressed IL-1ß stimulation of NFkB; however, upon reduction of BMP signaling through the addition of silencing RNA to BMPR2, this NFkB signaling was restored (Fig. 2e).

**Fig. 3.**
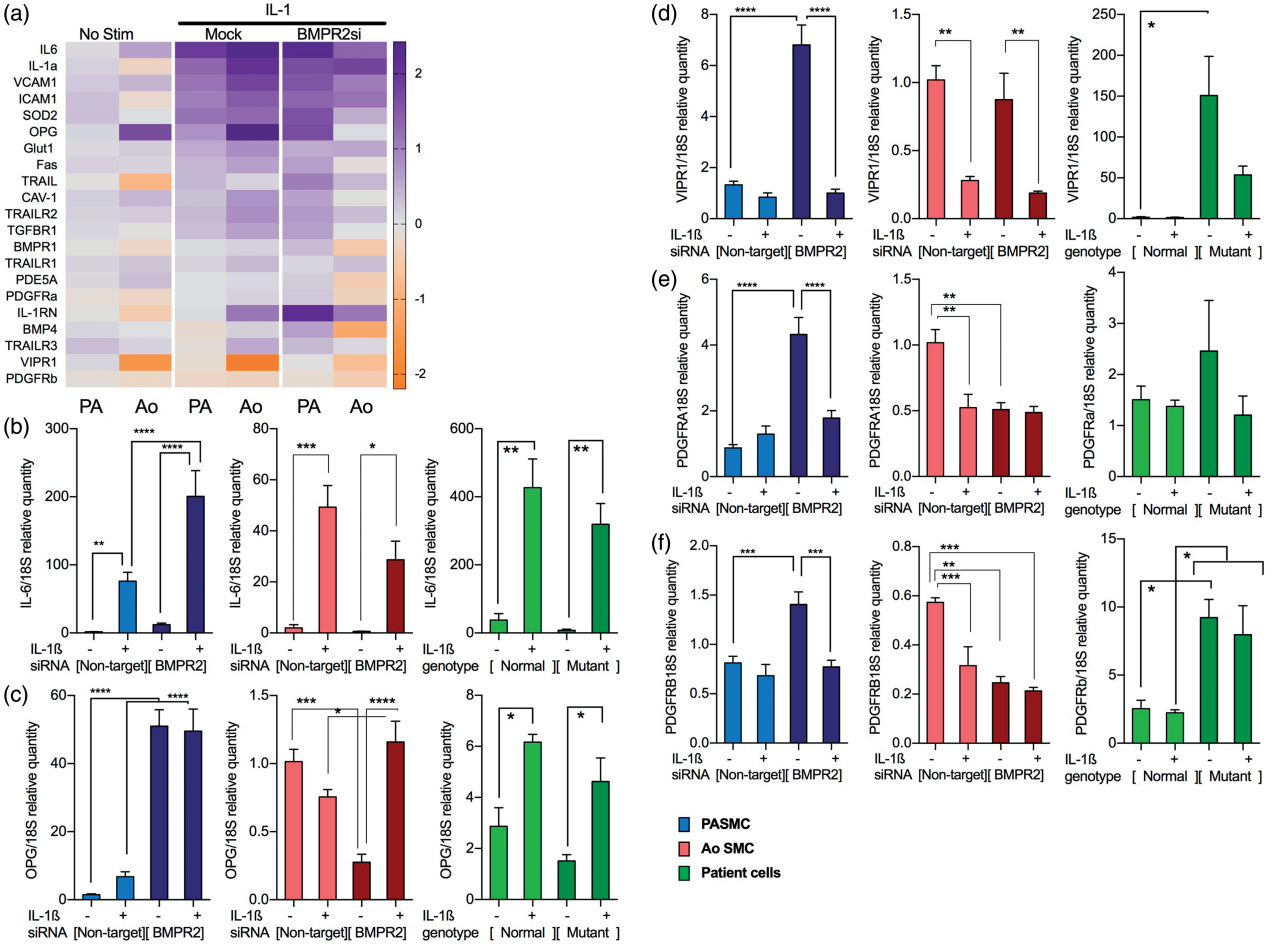
Comparison of the effect of reduced BMPR2 expression on IL-1-induced gene expression in commercial AoSMC and PASMC (a). IL-1ß stimulation in conditions of reduced BMPR2 expression in PASMC resulted in a greater induction of IL-6 compared to normal BMPR2 expression in PASMC but not in AoSMC or in PASMC taken from donors with and without known BMPR2 mutations (b). Expression of OPG is increased in PASMC upon loss of BMPR2 regardless of IL-1 ß stimulation. In AoSMC, BMPR2 loss causes a decrease of OPG which was reversed by IL-1ß stimulation; in donor cells increases were seen in OPG to IL-1ß stimulation regardless of their mutation status (c). VIPR1 expression is unaffected by loss of BMPR2 in aortic cells; however, PASMC lacking BMPR2 show baseline increases which normalize to IL-1ß. PASMC taken from donors display the same pattern as commercial PASMCs where loss of BMPR2 function induces a large increase in VIPR1 expression which can be normalized to an extent by IL-1ß (d). Array validation by qRT-PCR of receptor expression showed that expression of PDGF receptors in PASMC increased to loss of BMPR2 expression but normalized by IL-1ß; this trend is also the case in donor PAMSCs in PDGFRB, whereas PDGFRB is increased in mutant patient cells regardless of IL-1ß stimulation. In AoSMC, however, loss of BMPR2 induces loss of PDGFRa and b expression (e, f). Ordinary one-way ANOVA with multiple comparison by Tukey’s post-test (*P < 0.05, **P < 0.01, ***P < 0.001, ****P < 0.0001).

To consider the effects of reduced BMPR2 expression in PASMC, validation was performed on PASMC with and without transfection using silencing RNA to BMPR2. A small panel of genes was put together from a disease relevant gene list^13^ to validate the microarray data that would cover the range expression changes observed. Minimal transcriptional changes were noted in the mock transfection samples (data not shown); however, reduced BMPR2 expression combined with IL-1ß stimulation caused a further increase in expression of the pro-inflammatory genes within the panel. This is not the case in AoSMC with many genes being decreased upon reduced BMPR2 expression, a summary of all the TaqMan carried out has been included (Fig. 3a).

At the individual gene level, IL-1ß stimulation in conditions of reduced BMPR2 expression in PASMC resulted in a greater induction of IL-6 compared to normal BMPR2 expression in PASMC. Using the same regime, there was no change in IL-6 in aortic cells or in PASMC taken from donors with and without known BMPR2 mutations (Fig. 3b). Loss of BMPR2 also caused large increases in the expression of OPG regardless of IL-1 ß stimulation in PASMC, but gave rise to a decrease of OPG in unstimulated aortic cells which was reversed by IL-1ß stimulation, in donor cells increases were seen in OPG to IL-1ß stimulation regardless of their mutation status (Fig. 3c). VIPR1 in aortic cells is unaffected by loss of BMPR2 function; however, PASMC showed a significant increase at baseline which was normalized by IL-1ß. PASMC taken from donors display the same pattern as commercial PASMC, where loss of BMPR2 function induces a large increase in VIPR1 expression which can be normalized to an extent by IL-1ß (Fig. 3d).

Array validation by qRT-PCR of receptor expression showed that expression of PDGF receptors in PAMSC increased to loss of BMPR2 expression but normalized to IL-1ß stimulation. This trend is also the case in donor PAMSC in PDGFRA, whereas PDGFRB is increased in mutant patient cells regardless of IL-1ß stimulation. In AoSMC, however, loss of BMPR2 induces loss of PDGFRa and b expression (Fig. 3e and 3f).

### BMPR2 deficiency exaggerates inflammatory activation to IL-1ß stimulation in R899X^+/–^ BMPR2 transgenic mice fed high-fat diet

**Fig. 4.**
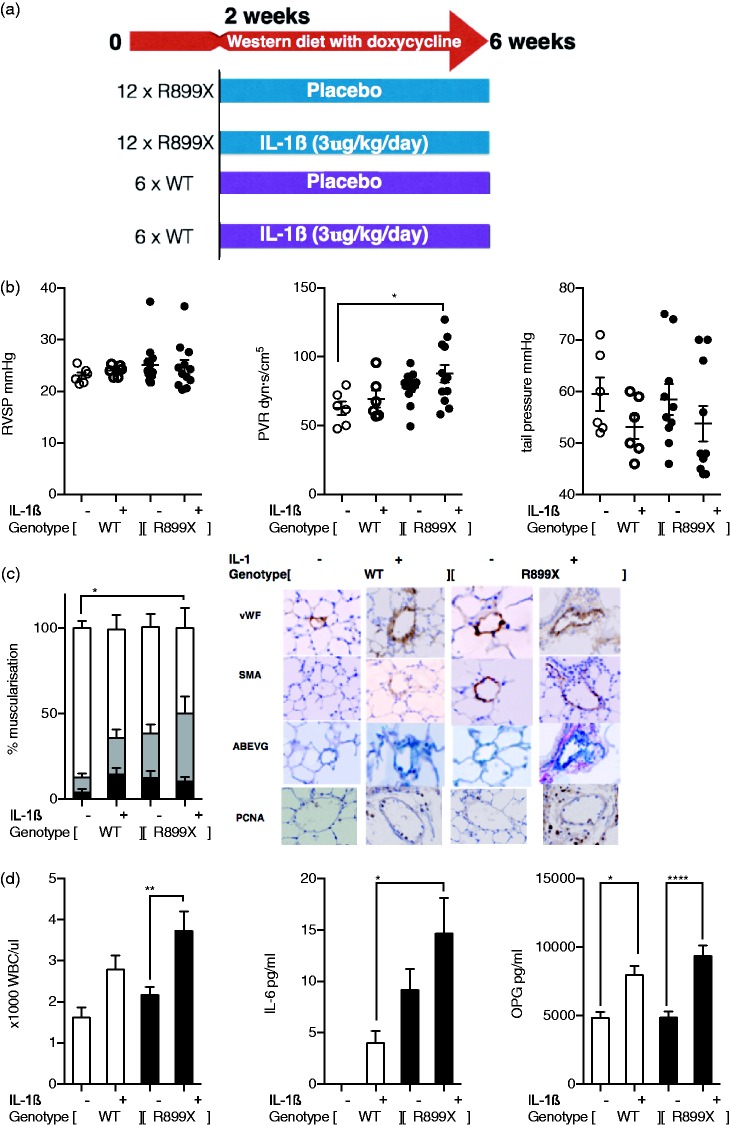
Disease model protocol for IL-1ß dosing (a). No significant changes were seen in RVSP, whereas calculated PVR shows significant increase upon disruption of BMPR2 signaling by mutant overexpression and IL-1ß stimulation along with increases in WBC count indicating a greater inflammatory response to IL-1ß in the mutant mice (b). Smooth muscle actin and PCNA content of the small arterioles increases with a combination of IL-1ß increase and BMPR2 loss with representative IHC images (c). Increases in serum levels of IL-6 are seen in the IL-1ß treated mutant mice compared to the wild-type litter-mates and serum levels of OPG display significant increase to IL-1ß regardless of mutation status (d). Ordinary one-way ANOVA with multiple comparison by Tukey’s post-test (*P < 0.05, **P < 0.01, ***P < 0.001, ****P < 0.0001)

To determine if these in vitro observations were relevant in vivo, we next investigated whether IL-1ß supplementation alters PAH phenotype in BMPR2 mutant mouse, overexpressing the mutation R899X in the BMPR2 gene. These mice have been previously shown to have disrupted BMPR2 signaling.^16^ In accordance with the protocol outlined in Fig. 4a, Rosa26-Bmpr2^R899X^ were given IL-1ß treatment. RVSP was unaffected; however, pulmonary vascular resistance (PVR) was increased in mutant mice treated with IL-1ß while systemic blood pressure was unaffected (Fig. 4b). Modest increases in small pulmonary arteriole muscularization and SMA- and PCNA-positive cells (Fig. 4c) suggesting more advanced pulmonary vascular remodeling were also seen. Delivery of IL-1 ß was evidenced by the increased white blood cell (WBC) counts. Serum levels of IL-6 were increased in mutant IL-1ß-treated Rosa26-Bmpr2^R899X^ animals. OPG was increased in response to IL-1ß, regardless of mutation status (Fig. 4d).

**Fig. 5.**
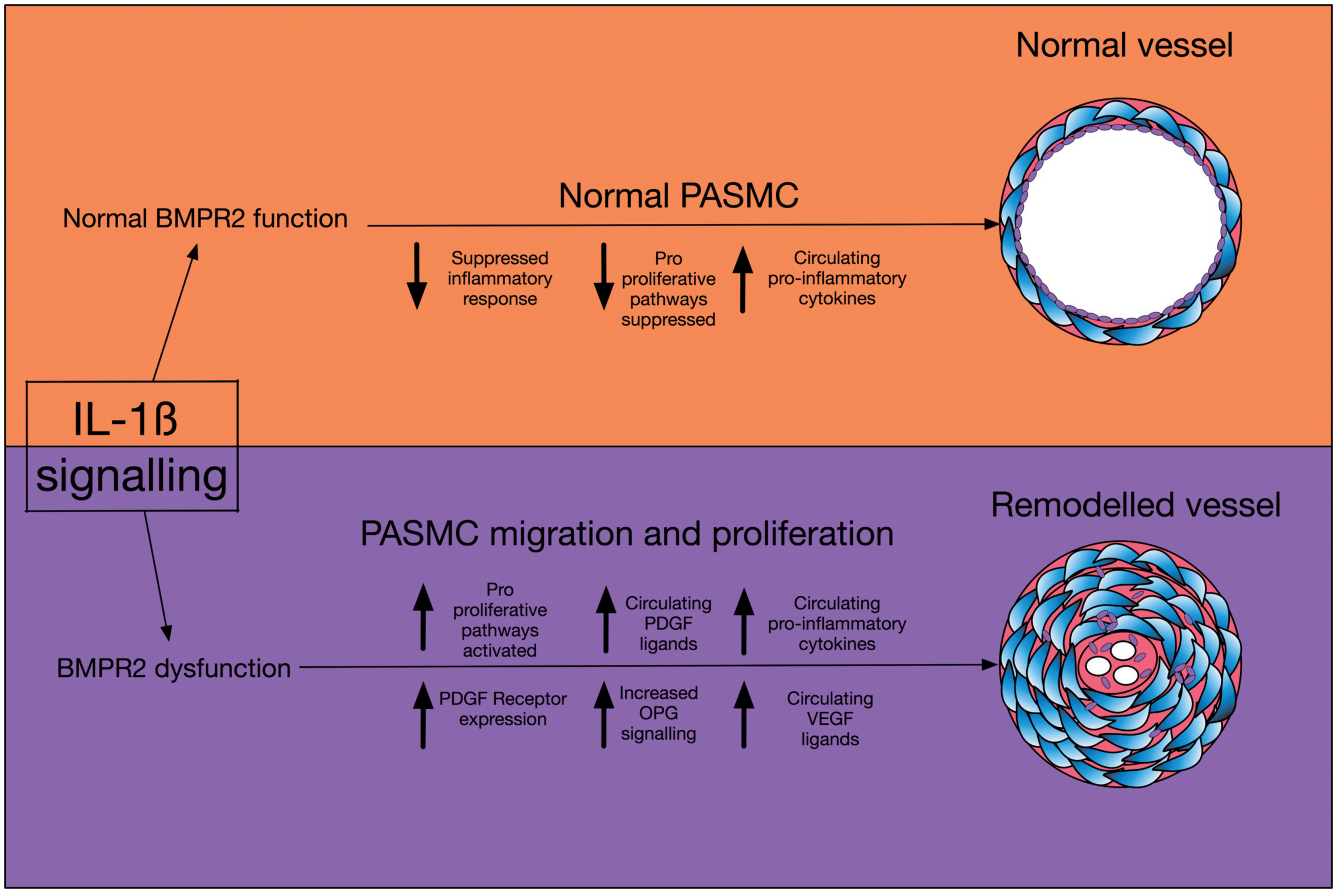
A schematic diagram of the pathways discussed in this paper.

## Discussion

Using unbiased microarray analysis, we report that IL-1ß induces vascular bed-specific transcriptional regulation in SMCs. Control (normal) PASMC display reduced inflammatory activation to IL-1ß compared with AoSMC in vitro. mRNA and pathway analysis identified a dampened pro-inflammatory, pro-proliferative response to IL-1ß in PASMCs compared to AoSMC which was lost upon loss of functional BMPR2. These findings are consistent with reports demonstrating the beneficial effects of IL-1 receptor antagonist (IL-1ra) in animal models of PAH where BMPR2 is lowered, such as the monocrotaline model.^9^ Furthermore, we demonstrate that BMPR2 signaling dysfunction results in increased inflammatory signaling and activation of mitogenic pathways such as PDGF, which are inhibited in normal PASMC in vitro (Fig 5), but this does not occur in SMCs from the systemic vascular bed. This is an inherent part of the disease pathogenesis and our findings could go some way to explain why patients with BMPR2 mutations go on to develop pulmonary vascular remodeling without effects on their systemic vessels.

These findings are confirmed in the cells from lung transplant patients with and without disease-causing BMPR2 mutations.

We report for the first time the additive effect for the loss of BMPR2 and administration of IL-1ß on the catalogue of transcriptional activity. Mutant BMPR2 expression combined with IL-1ß caused raised PVR associated with increased small pulmonary vessel muscularization and correlated positively with serum IL-6 levels.

Therapeutic agents targeting IL-1ß are in clinical use, of note anakinra, which has recently been trialed in therapeutic use in PAH.^18^ This gives reason for further investigation into the role IL-1ß may play in disease; late-stage clinical trials using biologics (canukinumab)^19^ in cardiovascular disease gives further emphasis for insights into this potential therapeutic area.

Our data suggest that targeting IL-1ß may be an effective therapeutic strategy for PAH treatment and that patient stratification according to IL-1ß responsive signals could be useful for identifying patients with an increased likelihood of treatment response. An advantage of targeting IL-1ß specifically is that this may mitigate against the risk of opportunistic infection by allowing other IL-1 to participate in host defence.^20^ Other recent research has shown that targeting inflammatory signaling in combination with increasing BMPR2 function is helpful in the treatment of PAH by the use in animal models of the drug FK506 (Tacrolimus)^21^ and a subsequent phase IIa clinical trial.^22^

Manipulation of inflammatory cell infiltration and endothelial cell apoptosis by antagonizing the BLT1 receptor of leukotriene LTB_4_ has also been shown to be effective in vivo,^23^ which backs up our findings that a combination of inflammation and BMPR2 dysfunction is important. Interestingly, the importance of neutrophil elastase (NE) in the pathobiology of PAH has been well recognized,^24^ and recent work demonstrating NE mediated release of IL-1ß^25^ may provide further evidence for a central role of IL-1ß biology in the pathogenesis of PAH, and highlights additional evidence for the potential use of NE inhibitors in PAH.

## References

[1] Conway EM Collen D Carmeliet P Molecular mechanisms of blood vessel growth. Cardiovasc Res 2001; 49: 507–521. 11166264 10.1016/s0008-6363(00)00281-9

[2] Guignabert C Tu L Girerd B et al. New molecular targets of pulmonary vascular remodelling in pulmonary arterial hypertension. Chest 2015; 147: 529–537. 25644906 10.1378/chest.14-0862

[3] Thompson AAR Lawrie A Targeting vascular remodeling to treat pulmonary arterial hypertension. Trends Mol Med 2017; 23(1): 31–45. 27989641 10.1016/j.molmed.2016.11.005

[4] Paulin R Courboulin A Meloche J et al. Signal transducers and activators of transcription-3/pim1 axis plays a critical role in the pathogenesis of human pulmonary arterial hypertension. Circulation 2011; 123: 1205–1215. 21382889 10.1161/CIRCULATIONAHA.110.963314 PMC3545712

[5] West J Austin E Fessel JP et al. 2015. Rescuing BMPR2 signalling axis in pulmonary arterial hypertension. Drug Discov Today 2015; 18: 1241–1245. 10.1016/j.drudis.2014.04.015 PMC4396626 24794464

[6] Humbert M Monti G Brenot F et al. Increases interleukin-1 and interleukin-6 serum concentrations in severe primary pulmonary hypertension. Am J Respir Crit Care Med 1995; 151(5): 1628–1631. 7735624 10.1164/ajrccm.151.5.7735624

[7] Steiner MK Syrkina OL Kolliputi N et al. Interleukin-6 overexpression induces pulmonary hypertension. Circ Res 2009; 104: 236–244. 19074475 10.1161/CIRCRESAHA.108.182014 PMC5482545

[8] Davies RJ Holmes AM Deighton J et al. BMP type II receptor deficiency confers resistance to growth inhibition by TGF-β in pulmonary artery smooth muscle cells: role of pro-inflammatory cytokines. Am J Physiol Lung Cell Mol Physiol 2012; 302: L604–L615. 22227206 10.1152/ajplung.00309.2011 PMC3311534

[9] Voelkel NF Tudor RM Interleukin-1 receptor antagonist inhibits pulmonary hypertension induced by inflammation. Ann N Y Acad Sci 1994; 725: 104–109. 8030981 10.1111/j.1749-6632.1994.tb39794.x

[10] Lawrie A Hameed AG Chamberlain J et al. Paigen diet-fed apolipoprotein E knockout mice develop severe pulmonary hypertension in an interleukin-1-dependent manner. Am J Pathol 2011; 179: 1693–1705. 21835155 10.1016/j.ajpath.2011.06.037 PMC3181351

[11] Soon E Crosby A Southwood M et al. Bone morphogenetic protein receptor type II deficiency and increased inflammatory cytokine production. A gateway to pulmonary arterial hypertension. Am J Respir Crit Care Med 2015; 192: 859–872. 26073741 10.1164/rccm.201408-1509OC PMC4613895

[12] Parpaleix A Amsellem V Houssaini A et al. Role of interleukin-1 receptor 1/MyD88 signalling in the development and progression of pulmonary hypertension. Eur Respir J 2016; 48(2): 460–483. 10.1183/13993003.01448-2015 27418552

[13] Rothman AMK Arnold N Pickworth J et al. MicroRNA-140-5p and SMURF1 regulate pulmonary arterial hypertension. J Clin Invest 2016; 126(7): 2495–2508. 27214554 10.1172/JCI83361 PMC4922709

[14] Hameed AG Arnold ND Chamberlain J et al. Inhibition of tumor necrosis factor-related apoptosis-inducing ligand (TRAIL) reverses experimental pulmonary hypertension. J Exp Med 2012; 209: 1919–1935. 23071256 10.1084/jem.20112716 PMC3478928

[15] Spiekerkoetter E Guignabert C Perez VdJ et al. S100A4 and BMP-2 co-dependently induce vascular smooth muscle cell migration via pERK and chloride intracellular channel 4 (CLIC4). Circ Res 2009; 105(7): 639. 19713532 10.1161/CIRCRESAHA.109.205120 PMC2818124

[16] Johnson JA Hemnes AR Perrien DS et al. Cytoskeletal defects in BMPR2-associated pulmonary arterial hypertension. Am J Physiol Lung Cell Mol Physiol 2012; 302: L474–L484. 22180660 10.1152/ajplung.00202.2011 PMC3311512

[17] Fessel JP Chem C Frump A et al. Interaction between bone morphogenetic protein receptor type 2 and estrogenic compounds in pulmonary arterial hypertension. Pulm Circ 2013; 3(3): 564–577. 24618541 10.1086/674312 PMC4070799

[18] Pilot study of the safety and efficacy of anakinra (recombinant human interleukin-1 receptor antagonist) in pulmonary hypertension. clinicaltrials.gov identifier NCT01479010.

[19] Ridker PM Thuren T Zalewski A et al. Interleukin-1β inhibition and the prevention of recurrent cardiovascular events: rationale and design of the Canakinumab Anti-inflammatory Thrombosis Outcomes Study (CANTOS). Am Heart J 2011; 162(4): 597–605. 21982649 10.1016/j.ahj.2011.06.012

[20] Dinarello CA Simon A Van Der Meer JWM Treating inflammation by blocking interleukin-1 in a broad spectrum of diseases. Nat Rev Drug Discov 2012; 11(8): 633–652. 22850787 10.1038/nrd3800 PMC3644509

[21] Spiekerkoetter E Tian X Cai J et al. FK506 activates BMPR2, rescues endothelial dysfunction and reverses pulmonary hypertension. J Clin Invest 2013; 123(8): 3600–3613. 23867624 10.1172/JCI65592 PMC3726153

[22] Spiekerkoetter E Sung YK Sudheendra D et al. Low-dose FK506 (Tacrolimus) in end-stage pulmonary arterial hypertension. Am J Respir Crit Care Med 2015; 192(2): 254–257. 26177174 10.1164/rccm.201411-2061LE PMC4532822

[23] Tian W Jiang X Tamosiuniene R et al. Blocking macrophage leukotriene B_4_ prevents endothelial injury and reverses pulmonary hypertension. Sci Transl Med 2013; 5(200): 200ra117. 10.1126/scitranslmed.3006674 PMC4016764 23986401

[24] Nickel NP Spiekerkoetter E Gu M et al. Elafin reverses pulmonary hypertension via caveolin-1 dependent bone morphogenetic protein signaling. Am J Respir Crit Care Med 2015; 191(11): 1273–1286. 25853696 10.1164/rccm.201412-2291OC PMC4476518

[25] Alfaidi M Wilson H Daigneault M et al. Neutrophil elastase promotes Interleukin-1ß Secretion from human coronary artery endothelium. J Biol Chem 2015; 290(40): 24067–24078. 26269588 10.1074/jbc.M115.659029 PMC4591798

